# Quantitative functional BOLD (qfBOLD): A combined gradient-echo and spin-echo framework for oxygen extraction fraction (OEF) mapping with functional MRI

**DOI:** 10.1177/0271678X261453807

**Published:** 2026-05-28

**Authors:** Antonio Maria Chiarelli, Lucie Chalet, Sara Pomante, Davide Di Censo, Alessandra Caporale, Emma Biondetti, Fabrizio Fasano, Domenico Zaca, Giulia Rocco, Manuela Carriero, Francesca Graziano, Elizabeth Jane Fear, Maria Eugenia Caligiuri, Richard Geoffrey Wise, Michael Germuska

**Affiliations:** 1 Department of Neurosciences, Imaging, and Clinical Sciences, University ‘G d’Annunzio’ of Chieti-Pescara, Chieti, Italy; 2 Institute of Advanced Biomedical Technologies (ITAB), University ‘G d’Annunzio’ of Chieti-Pescara, Chieti, Italy; 3 Siemens Healthcare, Camberley, UK; 4 Siemens Healthineers, Forchheim, Germany; 5 Siemens Healthcare SRL, Milan, Italy; 6 Department of Medical and Surgical Sciences, University Magna Graecia of Catanzaro, Catanzaro, Italy; 7 Neuroscience Research Center, University Magna Graecia of Catanzaro, Catanzaro, Italy; 8 Department of Radiology, University of California Davis Medical Center, Sacramento, CA, USA

**Keywords:** Calibrated functional (f)MRI, cerebral metabolic rate of oxygen (CMRO_2_), gradient-echo blood oxygen level dependent (BOLD) fMRI, hypercapnia, oxygen extraction fraction (OEF), spin-echo BOLD fmri

## Abstract

We present a novel framework for OEF mapping with MRI, based on temporal variations in gradient echo (GE) and spin-echo (SE) BOLD signals induced by isometabolic modulations in CBF. This approach, termed quantitative functional BOLD (qfBOLD), exploits dynamic variations in relaxation times rather than measuring baseline values as in qBOLD, thereby isolating deoxyhaemoglobin (dHb) effects. The interaction between dHb-induced extravascular field distortions and water diffusion allows for decoupling OEF and dHb-sensitive cerebral blood volume with a single modulation in brain physiology. Furthermore, the method avoids functional CBF measures via arterial spin labelling which is required by calibrated (c)fMRI. This advancement may enhance signal-to-noise ratio and spatiotemporal resolution, making qfBOLD applicable to both grey matter (GM) and white matter (WM). Monte Carlo simulations were used to investigate the method. In vivo feasibility assessment using a hypercapnic breath-holding task yielded OEF values of 37.0% ± 2.9% and 41.6% ± 2.9% in GM and WM, respectively, and significant correlations with cfMRI in GM (qfBOLD vs cfMRI *r* = 0.71, *p* < 10^−3^) and with relaxometry-based measures in the superior sagittal sinus (GM qfBOLD vs TRUST *r* = 0.51, *p* < 0.05, WM qfBOLD vs TRUST *r* = 0.61, *p* < 0.01). Future efforts will aim to improve the method’s accuracy by attenuating intravascular signals and by refining WM modelling.

## Introduction

Oxidative metabolism provides most of the brain’s energy.^1,2^ The brain lacks energetic reserves, and it depends on oxygen supply via cerebral blood flow (CBF).^2,3^ The brain’s use of oxygen is quantified by the cerebral metabolic rate of oxygen (CMRO_2_).^4–6^ Oxygen transfer must obey the principle of conservation of mass which states that CMRO_2_ equals the rate of exit of oxygen from the vascular compartment.^2^ This implies that CMRO_2_ is equal to the product of oxygen supply (itself product of CBF and arterial oxygen concentration (CaO_2_)) and the fraction of oxygen extracted (i.e. the oxygen extraction fraction (OEF)).^7^

Magnetic resonance imaging (MRI) can be used to quantify CMRO_2_, offering advantages over the gold-standard approach of ^15^O PET, including the avoidance of ionising radiation and the need for a cyclotron to make short-lived contrast agents.^8,9^ While different MRI techniques can quantify perfusion (e.g. arterial spin labelling (ASL)),^10^ measuring OEF is more challenging. It requires the estimation of deoxyhaemoglobin (dHb) concentration in blood ([dHb]) to infer venous saturation (SvO_2_) and OEF.^11^ OEF can be reliably measured in large veins, but such methods do not localise the site of oxygen extraction.^12,13^

In contrast, evaluation of [dHb] in microvasculature allows the generation of OEF maps but it is affected by two main limitations. Firstly, estimating [dHb] requires knowledge of the dHb-sensitive cerebral blood volume (CBV_dHb_).^14^ Secondly, measurements can be affected by non-blood susceptibility sources, such as iron and myelin. This is mainly a concern for methods such as quantitative blood oxygen level dependent (qBOLD) MRI and quantitative susceptibility mapping (QSM), which aim to quantify OEF from an evaluation of signal decay and phase changes with echo time (TE), respectively.^15–18^ Calibrated functional (cf)MRI addresses the latter problem by probing the temporal modulations in the transverse relaxation rate (*R*_2_*, relaxation time *T*_2_* = 1/*R*_2_*) related to dHb variations. It combines gradient echo BOLD signal changes (BOLD_GE_) and ASL functional recordings with isometabolic modulations of CBF to infer, at a given TE, the maximum BOLD_GE_ increase obtainable with complete removal of dHb.^19^ The most informative approach is dual cfMRI, which uses a complex protocol alternating between hypercapnia (a rise in CO_2_ in arterial blood increasing CBF) and hyperoxia (increased O_2_ in arterial blood) through gas inhalation, to decouple baseline OEF from CBV_dHb_ and thereby map GM CMRO_2_.^20–24^ The principal shortcomings of cfMRI methods are the practical complexity and the need to correlate BOLD and ASL perfusion modulations. ASL has low temporal resolution and temporal signal-to-noise ratio (tSNR). In practice, it is generally feasible only in GM and not in WM. These features limit the applicability of cfMRI methods.^21^

Here we present a novel fMRI framework that estimates baseline [dHb] and OEF by combining *T*_2_*-weighted BOLD_GE_ and *T*_2_-weighted spin echo BOLD (BOLD_SE_) fMRI concurrently acquired during modulation of CBF. The method estimates OEF with the selective sensitivity to dHb of cfMRI but with a simplified protocol that requires hypercapnia only, and without the need for concurrent ASL recordings. The absence of functional ASL should produce measurements with higher SNR with the possibility to increase spatiotemporal resolution. Moreover, the approach may also be applied to WM where ASL is challenging. We refer to the method as quantitative functional BOLD (qfBOLD), since it uses functional BOLD_SE_ and BOLD_GE_ signal modulations to infer relaxation-rate changes and quantify OEF, analogous to qBOLD in its use of SE and GE deoxyhaemoglobin-sensitive signal modelling. However, unlike qBOLD, which typically relies on baseline multi-TE signal behaviour to estimate absolute relaxation properties such as *T*_2_*, *T*_2_ and *T*_2_′ (1/*T*_2_′ = 1/*T*_2_* − 1/*T*_2_), qfBOLD exploits temporal fluctuations in BOLD_SE_ and BOLD_GE_ signals assessed at a single TE.

Here, we firstly provide a descriptive overview of the approach. Secondly, we introduce the qfBOLD analytical framework, where the simple but robust and interpretable Davis model of the BOLD signal is used.^19^ Thirdly, we report on the validation of the method based on Monte Carlo simulations involving a multi-compartmental model of the BOLD_SE_ and BOLD_GE_ signals, exploring effects related to vessel topology, contribution of the intravascular signal and experimental noise. Finally, we test the method in healthy human subjects. We implemented a tailored BOLD–ASL sequence to concurrently acquire BOLD_GE_, BOLD_SE_ and pseudo-continuous ASL (pCASL) data during a breath-holding task (breath-hold, BH) that induced CBF changes via hypercapnic modulation. The sequence was developed specifically to compare the qfBOLD method to a single cfMRI approach that we recently developed to map OEF.^7,25^ qfBOLD was also compared to independent macrovascular measures of OEF derived from the superior sagittal sinus (SSS, TRUST, *T*_2_ relaxation under spin tagging).^12^

## Methods

### Overview of qfBOLD

The qfBOLD method estimates OEF by combining *T*_2_*-weighted BOLD_GE_ and *T*_2_-weighted spin echo BOLD_SE_ fMRI acquired during modulation of CBF. The framework exploits the fact that, in the presence of microvasculature and water diffusion, both extravascular *R*_2_ (1/*T*_2_) and *R*_2_* (1/*T*_2_*) are linearly related to CBV_dHb_ but they scale non-linearly, and differently, with respect to [dHb].^26–28^ The effect is stronger for smaller vascular compartments, such as capillaries. Since the BOLD signal modulations are approximately proportional to relaxation rates changes (−Δ*R*_2_* for BOLD_SE_ and −Δ*R*_2_ for BOLD_SE_), they will be non-linear functions of changes in [dHb] (Δ[dHb]) and will instead preserve a linear behaviour with CBV_dHb_ and its changes. Crucially, since the BOLD_SE_ weighting has an increased sensitivity to diffusion effects compared to BOLD_GE_ and it primarily ‘senses’ the capillary compartment, the BOLD_SE_ signal change exhibits an accentuated supralinear dependence on ∆[dHb] compared to BOLD_GE_. This implies that the contribution of ∆[dHb] to the signals can be isolated by comparing the BOLD_GE_ and the BOLD_SE_ signal changes, specifically, by taking their ratio. Notably, the functions linking BOLD_SE_ and BOLD_GE_ modulations to Δ[dHb] are influenced by vessel topology (for a fixed echo time, TE, readout scheme and intravascular signal suppression).^29–31^ The sensitivity of the BOLD_SE_/BOLD_GE_ signal change ratio to vessel topology is particularly strong when large vessels (>100 µm) are considered.^32^ However, we speculate that the effect of ∆[dHb] on the BOLD_SE_/BOLD_GE_ signal change ratio is generally stronger than the effect induced by variability in the average vessel sizes within a voxel where only microvasculature is present. This is because capillary and venular topology as well as their volume ratio within each voxel are not expected to vary extensively.^33–35^

Importantly, analogously to cfMRI relying on hypercapnia, a modulation in CBF makes ∆[dHb] a function of baseline [dHb],^23^ implying that the comparison of the BOLD_SE_ and BOLD_GE_ modulations during vasodilation is sensitive to baseline venous saturation and OEF.^23^ When other mechanisms are used to alter vascular susceptibility, like gadolinium administration or hyperoxia, the method does not depend on baseline [dHb], thus becoming selectively sensitive to vessel topology (i.e. vessel size imaging).^36–39^ When using CBF modulations to introduce sensitivity to baseline [dHb], the CBF changes affect the BOLD signals in a non-linear manner. However, the non-linear contributions of CBF modulations differ only slightly between BOLD_GE_ and BOLD_SE_ signal changes. As a result, the ratio of BOLD_SE_ to BOLD_GE_ modulations is not only independent of CBV_dHb_, but also nearly independent of the extent of CBF modulation, removing the need for ASL measurement of CBF.

### qfBOLD analytical modelling

The rate of signal decay of BOLD_GE_ due to dHb within a voxel with microvasculature can be expressed, according to the Davis model, as^19^:

(1) $$R_{2}^{*}{|}_{dHb}=A_{GE}\cdot CBV_{dHb}\cdot {\left[dHb\right]}^{\beta_{GE}}.$$

Here, β _GE_ encodes the supralinear dependence of *R*_2_* on [dHb] and it depends (as *A*_GE_) on the MRI sequence (TE, readout scheme), water diffusion around microvessels, vascular topology (e.g. average vessel radius) and intravascular/extravascular contributions to the BOLD_GE_ signal. Values of β_GE_ ≈ 1.3–1.5 have been reported in the literature for BOLD_GE_ at 3 T with TE_GE_ ≈ 30 ms.^7,19^ The net supralinear behaviour primarily arises from a linear large vessel component combined with supralinear contributions from capillaries to the extravascular relaxivity.^7,19^

In the presence of small ∆ $R_{2}^{*}$ induced by dHb modulation, the BOLD_GE_ signal change can be expressed as^19,22^:

(2) $$\begin{array}{l} \frac{\Delta BOLD_{GE}}{BOLD_{GE}}=-TE_{GE}\cdot \Delta R_{2}^{*}=\\ M_{GE}\cdot \left\{1-\left(\frac{CBV_{\mathrm{dHb},m}}{CBV_{dHb}}\right)\cdot {\left(\frac{\left[{\mathrm{dHb}}_{m}\right]}{\left[\mathrm{dHb}\right]}\right)}^{\beta_{GE}}\right\} \end{array}$$

where the subscript m indicates a modulation relative to the baseline value and M_GE_ is the maximum BOLD_GE_ signal change that can be obtained with complete removal of dHb. M_GE_ depends on baseline quantities and is equal to:

(3) $$M_{GE}=TE_{GE}\cdot A_{GE}\cdot CBV_{dHb}\cdot {\left(\left[dHb\right]\right)}^{\beta_{GE}}$$

$CBV_{\mathrm{dHb}}$ changes can be expressed as a function of CBF modulations via the Grubb relation,^41,42^ whereas [dHb] changes can be expressed as a function of CBF and CMRO_2_ changes obtaining:

(4) $$\frac{\Delta BOLD_{GE}}{BOLD_{GE}}=M_{GE}\cdot \left\{1-{\left(\frac{{\mathrm{CBF}}_{m}}{\mathrm{CBF}}\right)}^{\alpha -\beta_{GE}}\cdot {\left(\frac{{\mathrm{CMRO}}_{2,m}}{{\mathrm{CMRO}}_{2}}\right)}^{\beta_{GE}}\right\}$$

with α the Grubb exponent (α = 0.18).^41^

Assuming isometabolic vasodilation, equation (4) becomes:

(5) $$\begin{array}{l} \frac{\Delta BOLD_{GE}}{BOLD_{GE}}=M_{GE}\cdot \left\{1-{\left(\frac{{\mathrm{CBF}}_{m}}{\mathrm{CBF}}\right)}^{\alpha -\beta_{GE}}\right\}=\\ M_{GE}\cdot \left\{1-{\left(1+\frac{\Delta \mathrm{CBF}}{\mathrm{CBF}}\right)}^{\alpha -\beta_{GE}}\right\} \end{array}$$

If flow changes are of limited size, equation (5) can be linearised thereby obtaining^42^:

(6) $$\frac{\Delta BOLD_{GE}}{BOLD_{GE}}=M_{GE}\cdot \left(\beta_{GE}-\alpha \right)\cdot \frac{\Delta CBF}{CBF}$$

The refocussing pulse in a BOLD_SE_ sequence reduces the effect of dHb on the BOLD signal while enhancing BOLD signal sensitivity to water diffusion and capillary extravascular effects.^43^ Within the Davis model framework, the refocussing effect may be conceived as lowering the multiplicative constant A and increasing the exponent factor β. Consequently, we can derive a comparable set of equations for BOLD_SE_ related to small perturbations of *R*_2_ due to flow changes, using different values of A and β compared to BOLD_GE_, resulting in:

(7) $$\frac{\Delta BOLD_{SE}}{BOLD_{SE}}=M_{SE}\cdot \left(\beta_{SE}-\alpha \right)\cdot \frac{\Delta CBF}{CBF}$$

With *M*_SE_ equal to:

(8) $$M_{SE}=TE_{SE}\cdot A_{SE}\cdot CBV_{dHb}\cdot {\left(\left[dHb\right]\right)}^{\beta_{SE}}$$

By comparing BOLD_SE_ to BOLD_GE_ signal changes we obtain:

(9) $$\begin{array}{l} \frac{\frac{\Delta BOLD_{SE}}{BOLD_{SE}}}{\frac{\Delta BOLD_{GE}}{BOLD_{GE}}}=\frac{TE_{SE}\cdot A_{SE}\cdot \left(\beta_{SE}-\alpha \right)}{TE_{GE}\cdot A_{GE}\cdot \left(\beta_{GE}-\alpha \right)}\cdot \\ {\left(\left[dHb\right]\right)}^{\beta_{SE-GE}}=C\cdot {\left(\left[dHb\right]\right)}^{\beta_{SE-GE}} \end{array}$$

where *C* is a multiplicative constant and $\beta_{SE-GE}$ (value larger than 0) is the exponent representing the difference in βs between BOLD_SE_ and BOLD_GE_.

Hence, the BOLD_SE_/BOLD_GE_ signal change ratio can be assumed to be independent of baseline CBV_dHb_ and CBF modulations and, importantly, is a monotonic function of [dHb]. SvO_2_ and OEF can be derived from [dHb] based on the equations^7^:

(10) $$\left[\mathrm{dHb}\right]=\left(1-S_{v}O_{2}\right)\cdot \left[\mathrm{Hb}\right]$$

with [Hb] the haemoglobin concentration in blood, and:

(11) $$OEF=1-\frac{\varphi \cdot \left[Hb\right]}{CaO_{2}}\cdot S_{v}O_{2}$$

where ϕ is the oxygen binding capacity of haemoglobin (ϕ = 1.34 ml/g) and CaO_2_ is the oxygen concentration in arterial blood.

Notably, *C* and $\beta_{SE-GE}$ may be modified by vessel topology and intravascular signal contribution to the total BOLD signals even for fixed TEs and readout schemes. These factors necessitate further investigation of the proposed method beyond analytical modelling. We examined the validity of the modelling and the values of the two constants in equation (9) based on Monte Carlo simulations of the BOLD signal (see the Simulations section for further details).

### Simulations

#### Multi-compartmental Monte Carlo modelling of the BOLD signal

Similar to the work of Uludağ et al.,^44^ we implemented a Monte Carlo three-compartment model of the BOLD signal, which comprises venous CBV (CBV_v_), capillary CBV (CBV_cap_) and tissue volume (1 − CBV_dHb_), where CBV_dHb_ is the sum of CBV_v_ and CBV_cap_. The total MRI signal can then be expressed as:

(12) $$\begin{array}{l} S_{tot}=\left(1-CBV_{dHb}\right)\cdot S_{ex}+\left(1-In_{sup}\right)\cdot \\ \left(CBV_{v}\cdot S_{in,v}+CBV_{cap}\cdot S_{in,cap}\right) \end{array}$$

were *S*_ex_ is the extravascular signal and *S*_in,v_ and *S*_in,cap_ are the intravascular signals for the venous and capillary compartment, respectively. Importantly, since the proposed approach exploits the extravascular BOLD signals, its performance is affected by the extent of intravascular signal, which can be suppressed using tailored sequences. Thus, the parameter In_sup_ in equation (12) was introduced to depict the level of intravascular suppression (ranging between 0 and 1). As in previous work by our group,^7^ the relation between CBV_dHb_, CBV_v_ and CBV_cap_ was assumed to be modulated by a parameter ρ, such that:

(13) $$\{\begin{array}{c} CBV_{dHb}=\rho \cdot CBV_{cap}\\ CBV_{v}=(\rho -1)\cdot CBV_{cap} \end{array}$$

Notably, ρ = CBV_dHb_/CBV_cap_ modulates the capillary and venular contribution to the BOLD signal and, as such, it also modulates the average vessel radius within the voxel.

An expected value of ρ = 2 was assumed^7^ with a certain level of variability allowed.

The GE and SE signals for each compartment were assumed equal to:

(14) $$\{\begin{array}{c} S_{GE}=S_{o}\cdot e^{-R_{2}^{*}\cdot TE_{GE}}\\ S_{SE}=S_{o}\cdot e^{-R_{2}\cdot TE_{SE}} \end{array}$$

with TE_GE_ and TE_SE_ = 30 and 85 ms, respectively (matching our in-vivo recordings).

Monte Carlo modelling within the ODIN framework^45^ was used to simulate extravascular *R*_2,ex_* and *R*_2,ex_ as a function of CBV_dHb_ and magnetic susceptibility χ for venules and capillaries.^44^ Radiuses of 5 and 24 μm were chosen to reflect the capillary and the venular scale, respectively. The vasculature was modelled as fully permeable to water, randomly oriented, cylinders of infinite length.

To quantify the [dHb] contribution to the extravascular signal, χ was calculated from vessel oxygen saturation, SO_2_ as^44^:

(15) $$\Delta \chi =4\pi \cdot Hct\cdot \Delta \chi_{\mathrm{do}}\cdot \left(\left|{\mathrm{SO}}_{2,\mathrm{off}}-{\mathrm{SO}}_{2}\right|\right)$$

where Δχ is the susceptibility difference between the vessel and the surrounding tissue, Δχ_do_ = 0.264 ppm is the susceptibility of fully deoxygenated blood,^45^ Hct is the haematocrit (computed assuming a proportionality with [Hb], Hct/[Hb] = 3%dl/g).^46^ SO_2,off_ was assumed to be SO_2,off_ = 0.95.^45^

Random vessel orientations were accounted for by varying the angle between the main field and the cylinder in the range from 0 to π/2 (in 64 linear steps). A sinθ weighting was applied to the signal values to account for the density of possible vessel angles with respect to the static field. For each simulated radius and vessel orientation, 10^5^ random walks were simulated from randomly seeded starting points. The temporal step size of each simulation was 1 ms, with the displacement at each step generated by a gaussian distribution to represent isotropic diffusion.

Diffusion was assumed to be 0.76 μm^2^/ms, as is typical in GM measurements,^47,48^ and a CBV_dHb_ = 3% was used for explicit simulations and assumed to have a linear effect on relaxation rates when a different blood volume was required. Gradient echo times of 17, 32.5 and 48 ms were used to simulate GE signals which were monoexponentially fitted to calculate *R*_2,ex_* values, while a mono-exponential fit from the initial amplitude and a spin echo at 85 ms was used to estimate *R*_2,ex_.

The total extravascular *R*_2_* and *R*_2_ were calculated from a linear combination of a fixed non-blood relaxation rate arising from the tissue and the extravascular contributions from the venous and capillary blood compartments:

(16) $$\{\begin{array}{c} R_{2,ex}^{*}=R_{2,ex,t}^{*}+R_{2,ex,v}^{*}+R_{2,ex,cap}^{*}\\ R_{2,ex}=R_{2,ex,t}+R_{2,ex,v}+R_{2,ex,cap} \end{array}$$

Tissue $R_{2,ex,t}^{*}$ and $R_{2,ex,t}$ were assumed equal to 21 and 13 Hz at 3 T,^44^ respectively. The extravascular GE and SE signals were calculated from *R*_2_*,_ex_ and *R*_2,ex_ according to equation (14).

Intravascular *R*_2_* and *R*_2_ for veins and capillaries were derived from the phenomenological equations reported by Zhao et al. and used to estimate intravascular signals from equation (14).^49^ Capillaries were assumed to have an oxygen saturation midway between arteries and veins. Intravascular signals (in veins and capillaries) were combined with the extravascular signals based on equation (12) to obtain total gradient and spin echo signals (*S*_tot_GE_ and *S*_tot_SE_).

During vasodilation and modulation of CBF, the relative CBV change for the two intravascular compartments was assumed to follow the Grubb relation (α = 0.18),^41^ whereas the [dHb] was selectively affected by CBF changes (constant CMRO_2_). The BOLD_GE_ and BOLD_SE_ signal changes were computed as:

(17) $$\{\begin{array}{c} \frac{\Delta BOLD_{GE}}{BOLD_{GE}}=\frac{S_{tot\_GE,m}-S_{tot\_GE}}{S_{tot\_GE}}\\ \frac{\Delta BOLD_{SE}}{BOLD_{SE}}=\frac{S_{tot\_SE,m}-S_{tot\_SE}}{S_{tot\_SE}} \end{array}$$

where the subscript tot_GE and tot_SE represent the total gradient and spin echo signals, respectively, and *m* represents the signal arising from vasodilatory modulation. Notably, from now on, we will refer to the total BOLD_GE_ and BOLD_SE_ signal changes (left-hand side of equation (17)) simply as BOLD_GE_ and BOLD_SE_.

#### Forward and inverse model simulations

To address the method’s sensitivity to confounding factors and the validity of the Davis model, we ran forward and inverse modelling simulations by exploiting the Monte Carlo modelling of the BOLD signal. The physiological parameters affecting the BOLD_GE_ and BOLD_SE_ changes, such as [Hb], CaO_2_, OEF, flow modulation (ΔCBF/CBF) and ρ were varied as random variables spanning plausible physiological ranges.

**Figure 1.**
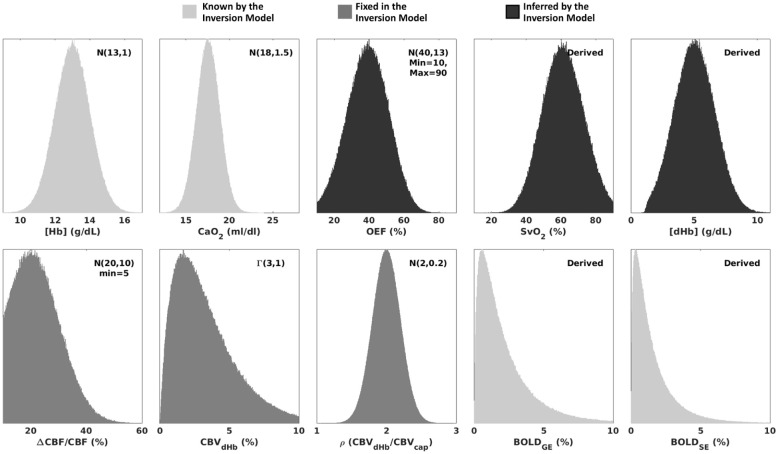
Random variables used to perform the multi-compartmental Monte Carlo forward and inverse model simulations. The variables reported in light grey were assumed to be known by the inversion model, those reported in medium grey were fixed a priori in the inversion model and those in dark grey were inferred by the inversion model.

The inversion model was implemented using two strategies: (1) using a grid search approach applied to Monte Carlo modelling (OEF: 0–1, 10^−3^ step, fixed ΔCBF/CBF = 20%, CBV_dHb_ = 3% and ρ = 2) and (2) explicitly inverting the Davis model (equations (9)–(11)), with parameters fitted using forward Monte Carlo simulations. Figure 1 illustrates the main simulated parameters and their corresponding probabilistic distributions. Notably, the varying parameters used to compute the forward model were either assumed to be known (i.e. [Hb], CaO_2_, BOLD_GE_, BOLD_SE_) or were fixed in the inversion model (ΔCBF/CBF, CBV_dHb_, ρ), while the remaining were inferred (OEF, SvO_2_ and [dHb]).

The effects of intravascular signal suppression (In_sup_), variability in ρ and experimental noise on the inversion model performance were also explored. For the latter, white gaussian noise was added to BOLD signal changes, and SNR was calculated as the expected signal change with the increase in blood flow normalised by noise standard deviation.

### Participants

Twenty-eight healthy subjects (19 males; age, mean ± standard deviation: 31 ± 8 years) were enrolled, after providing written informed consent. The study was performed in accordance with the Declaration of Helsinki and approved by the Institutional Review Board (protocol number 14-C, 11/12/2023) of the Department of Neurosciences, Imaging and Clinical Sciences, University ‘G d’Annunzio’ of Chieti-Pescara, Italy.

### MRI acquisition

Data were acquired at the Institute for Advanced Biomedical Technologies, University ‘G d’Annunzio’ of Chieti-Pescara, Italy, on a 3 T MAGNETOM Prisma scanner (Siemens Healthineers AG, Forchheim, Germany), featuring a 32-channel receive-only head coil. The fMRI measurements were conducted with a customised pCASL research sequence. An BOLD-ASL sequence was developed based on a manufacturer’s product sequence utilising a dual-excitation (DEXI) echo planar imaging (EPI) 2D readout that includes a short TE, TE1 = 10 ms for ASL, and, following a second excitation, a longer TE, TE2 = 30 ms for BOLD_GE_.^7,51^ The separate readouts enabled optimal background suppression during the ASL image acquisition while allowing a sufficient amount of longitudinal magnetisation recovery for BOLD readout. Furthermore, a refocussing pulse was added after the second excitation for each slice to generate SE weighting, with TE3 = 85 ms. Pre-labelling saturation and background suppression were included.^52^ The ASL labelling duration (τ) and post-labelling delay (PLD) were set to 1.5 s, and GRAPPA acceleration was applied (factor = 3). An effective TR of 5 s was utilised to acquire 14 slices, with in-plane resolution of 3.4 × 3.4 mm^2^ and slice thickness = 7 mm, with a 30% slice gap for full brain coverage.

fMRI data were collected during a BH task. The BH protocol included a baseline period of 60 s followed by 10 cycles of post-expiratory breath-holding, each lasting 20 s, interleaved with a 40 s-normal breathing, totalling 11 min and 28 s.^25^ Subjects were visually cued with instructions executed using E-Prime 3.0 stimulus presentation software (Psychology Software Tools, Pittsburgh, PA, USA). Participants were directed to maintain a neutral diaphragm position during BH and to completely exhale at the end of each BH.^53^

During the fMRI recordings, CO_2_ partial pressure in the exhaled air was measured using a nasal cannula connected to a gas analyser system (ADInstruments, Dunedin, New Zealand).

Two proton density images (*S*_0_) were obtained for susceptibility distortion correction and ASL calibration with pCASL labelling and background suppression pulses turned off, with TR = 7 s and TE = 10 ms, and opposite phase encoding directions. A MP2RAGE *T*1-weighted scan was conducted for registration and brain segmentation purposes (matrix 176 × 256 × 256, 1 mm isotropic resolution, TR/TE = 5000/3.58 ms, TI1/TI2 = 700/2500 ms).^53^ In a subgroup of 16 subjects, blood *T*_2_ in the SSS was estimated from TRUST acquisition (inversion time = 1020 ms, $\tau_{CPMG}$ = 10 ms, labelling thickness 100 mm and *T*_2_-prepared effective (e)TEs 0, 40, 80 and 160 ms).^12^ Please refer to Table S1 in Supplementary Materials for additional information about MRI sequences.

### Data processing

#### Gas recordings pre-processing

End-tidal O_2_ and CO_2_ partial pressures (PetO_2_ and PetCO_2_), taken as a surrogate measure of arterial oxygen (PaO_2_) and CO_2_ (PaCO_2_) partial pressures, were extracted using in-house software in MATLAB (version R2022b).^7,54^

The PetO_2_ and PetCO_2_ traces were calculated by isolating the minima (for the O_2_) and the peaks (for CO_2_) of the traces from the gas analyser. The end-tidal signals were resampled at the fMRI TR and shifted to account for time lags between expiration and recordings. Baseline PaO_2_ and PaCO_2_ values were estimated in the first 60 s.

Baseline PaO_2_ was used to infer arterial oxygen saturation, SaO_2_, through the equation^42^:

(18) $$SaO_{2}=\frac{1}{1+{\left(\frac{P_{50}}{PaO_{2}}\right)}^{h}}$$

where *h* is the Hill constant (*h* = 2.8) and P_50_ is oxygen pressure when haemoglobin is half saturated (P_49_ = 26 mmHg). CaO_2_ was inferred from^7^:

(19) $$CaO_{2}=\varphi \cdot \left[Hb\right]\cdot SaO_{2}+\varepsilon \cdot PaO_{2}$$

where ε is the oxygen plasma solubility (ε = 0.0031 ml/mmHg/dl). [Hb] was assumed to be 13 g/dl.

PetCO_2_ traces were band-pass filtered (Butterworth digital filter, cut-off times of 10 and 150 s), to be used as regressor to estimate BOLD_GE_, BOLD_SE_ and ASL perfusion signal changes in response to the BH stimulus.

#### Anatomical MRI and fMRI pre-processing

The MP2RAGE UNI image was employed for tissue segmentation (FAST, FSL) and for warping into MNI space (antsRegistration, SyN, ANTs).^56–58^

The pre-processing steps of fMRI data involved data normalisation, motion correction, susceptibility distortion correction and filtering and were performed using FSL, ANTs and in-house MATLAB algorithms.^55,56^ The two proton density (*S*_0_) images were corrected for susceptibility distortions (Topup, FSL)^58^ and intensity inhomogeneity (N4biasfieldcorrection, ANTs).^59^ The corrected *S*_0_ image, skull-stripped using FSL BET, was rigidly registered to the *T*1-weighted image, and the transformation matrix was inverted to bring the GM and WM partial volume estimates into the *S*_0_ space. These were thresholded (th = 0.5) to obtain compartmental masks. An fMRI motion correction pipeline was applied to the fMRI BH data (Supplementary Materials).^60^ The motion corrected volumes were then rigidly registered (ANTs) to the brain-extracted *S*_0_, acquired with the same phase encoding direction as the functional scans. The fMRI volumes for each echo were then corrected for susceptibility distortions (FSL ApplyTopup).

The perfusion signals (∆*S*) in *S*_0_ space were obtained through surround subtraction of the fMRI timecourses at TE1^10^ and converted to CBF in quantitative units of ml/100 g/min through the pCASL single compartment kinetic model^10^:

(20) $$CBF=\frac{6000\cdot \lambda \cdot e^{\frac{PLD}{T1_{b}}}}{2\cdot \eta \cdot \eta_{inv}\cdot T1_{b}\cdot \left(1-e^{-\frac{\tau }{T1_{b}}}\right)}\cdot \left(\frac{\Delta S}{S_{0}}\right)$$

with λ the water partition coefficient (λ = 0.9 ml/g), *T*1_b_ the *T*1 of arterial blood (*T*1_b_ = 1.67 s), η the tagging inversion efficiency (η = 0.85) and η_inv_ a scaling factor accounting for reduction in tagging efficiency due to background suppression (η_inv_ = 0.88).^62^

Surround averaging was performed on the BOLD signals to eliminate ASL contamination. CBF and BOLD signals were expressed as relative changes with respect to the baseline. All three fMRI signals were band-pass filtered in analogy with the PetCO_2_ trace.

#### qfBOLD, cfMRI and TRUST data processing

Processing was performed in MATLAB. The evaluation of voxel-wise BOLD and CBF modulation in response to the BH task was performed using linear regression,^62^ where the filtered PetCO_2_ trace served as the independent variable. The filtered PetCO_2_ was allowed to shift by ±10 s to account for haemodynamic lags.^25^ The regression provided estimates of cerebrovascular reactivity (CVR, signal change per unit of PetCO_2_ change), which were multiplied by a metric of modulation in the PetCO_2_ trace (the difference between the 95th percentile and the fifth percentile of the signal) to obtain BOLD and CBF signal changes in response to the BH tasks.

qfBOLD analysis to infer OEF maps was performed based on voxel-wise ratio of the BOLD_SE_ and the BOLD_GE_ signal changes in response to the BH task. This data was input into an inversion model based on the Davis model (equations (9)–(11)) which had been fitted to Monte Carlo simulations (please refer to the Results section). For within-session repeatability assessment, the same processing was performed by dividing the filtered traces by considering separately the first five BH and the last five BH.

A cfMRI analysis was also performed based on the comparison of the BOLD_GE_ and ASL signal modulations in response to BH and by employing a single-calibration framework we developed to infer OEF in the GM.^7,25^ The approach is described in detail in Chiarelli et al.,^7^ but essentially infers OEF from the maximum BOLD_GE_ modulation (M_GE_).^7,25^ Briefly, using the central volume theorem, the model expresses the baseline CBV_dHb_ as the product of CBF (that we measure at baseline with ASL) and the mean transit time (MTT) within the compartment of interest to then link the MTT with OEF via the flow-diffusion model of oxygen transport, thus solving the problem of decoupling the dependence of M_GE_ on CBV_dHb_ and OEF. The model requires the assumption of a negligible oxygen tension at the mitochondria, which is plausible in the healthy brain. The equation relating M_GE_ to OEF is^7,25^:

(21) $$\begin{array}{l} M_{GE}=TE_{GE}\cdot \frac{A_{GE}\cdot \rho }{k}\cdot \\ \frac{CBF\cdot OEF\cdot CaO_{2}\cdot {\left(\left(1-\frac{CaO_{2}}{\varphi \left[Hb\right]}\cdot \left(1-OEF\right)\right)\cdot \left[Hb\right]\right)}^{\beta_{GE}}}{\left(P_{50}\cdot \sqrt[{h}]{\frac{2}{OEF}-1}\right)} \end{array}$$

were *k* is the effective oxygen permeability of brain tissue. Equation (5) was used to estimate M_GE_ and equation (21) was inverted to estimate the OEF using a grid search approach (OEF between 0 and 1, in steps of 10^−3^). We assigned a value of 1.3 to β_GE_ and a value of 8.8/s/g^β^dl^β^/(μmol/mmHg/ml/min) to the term (*A*_GE_ ρ)/*k*, matching our previously established in-vivo estimations.^7^

Global venous *T*_2_ and OEF was also estimated from the TRUST acquisition through the fitting of signal decay with effective TE in the SSS and a calibration model.^63^

### Statistical analysis

Statistical analysis was performed in MATLAB. Root mean square errors (RMSEs) and Pearson’s correlations were evaluated to assess associations between the variables of interest. *T*-tests were conducted to evaluate statistical significance. A *p* < 0.05 was considered significant.

## Results

### Simulations

**Figure 2.**
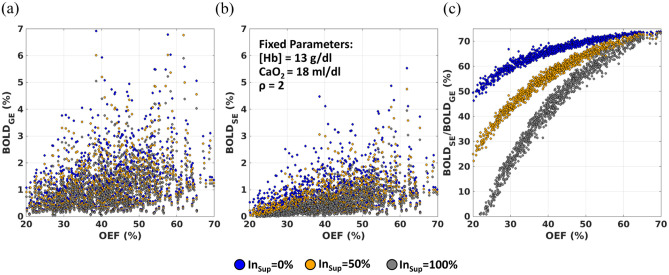
Forward model simulations when fixing [Hb], CaO_2_ and ρ (ρ = CBV_dHb_/CBV_cap_). The images show (a) BOLD_GE_ modulation, (b) BOLD_SE_ modulation and (c) BOLD_SE_/BOLD_GE_ signal change ratio as a function of OEF, for three levels of intravascular signal suppression (0%, 50% and 100%).

Figure 2 shows the results of forward model simulations when fixing [Hb], CaO_2_ and ρ. Results for vascular suppression of 0%, 50% or 100% are reported. Notably, when [Hb] and CaO_2_ are fixed, the OEF is linearly related to [dHb] (from which OEF is derived through equations (10) and (11) and to which BOLD methods are sensitive). Figure 2(a) and (b) show the BOLD_GE_ and the BOLD_SE_ signal as a function of OEF. As expected, no significant association between OEF and BOLD signals was found due to variability in baseline CBV_dHb_ and in ΔCBF/CBF (all other parameters except OEF were fixed). On the contrary, when evaluating the BOLD_SE_/BOLD_GE_ signal changes ratio, a clear monotonic dependence on OEF is visible with reduced effects introduced by baseline CBV_dHb_ and ΔCBF/CBF. Importantly, the rate of monotonic dependance is higher for stronger intravascular signal suppression. A larger suppression of the intravascular BOLD signal also tends to decrease the BOLD_SE_/BOLD_GE_ signal changes ratio for a given OEF value. Based on average experimental values of the BOLD_SE_/BOLD_GE_ signal changes ratio in GM and average global OEF estimation from TRUST, we inferred that our BOLD_GE_/BOLD_SE_ ASL sequence showed an intravascular suppression of BOLD signals ~50% (In_sup_ = 0.5 in equation (12)).

**Figure 3.**
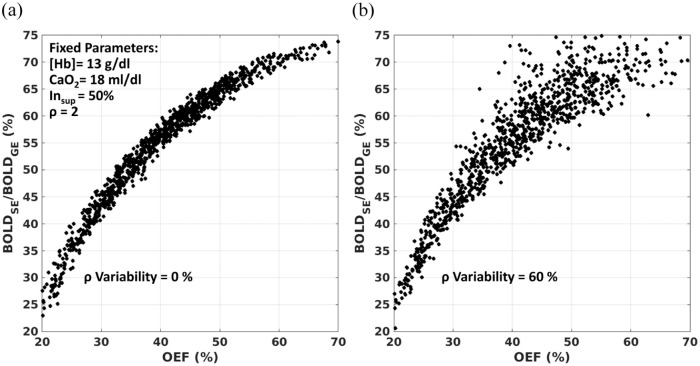
Forward model simulations when fixing [Hb], CaO_2_ and In_sup_ (In_sup_ = level of intravascular signal suppression, between 0 and 1). The subplots show BOLD_SE_/BOLD_GE_ signal change ratio as a function of OEF for (a) ρ variability (±3 σ/μ) = 0% and (b) ρ variability = 60%.

Reports forward model simulations as in Figure 2 with an intravascular suppression of 50%, and ρ variability (±3 σ/μ) of 0% (Figure 3(a)) and 60% (Figure 3(b)). As ρ modulates the ratio between capillary and venules, which differently affect BOLD_GE_ and BOLD_SE_, the variability in ρ is reflected in a higher noise introduced in the BOLD_SE_/BOLD_GE_ monotonic relation with OEF. We fitted the Davis model (equation (9)) to the data in Figure 3(a) around expected values of OEF (0.25–0.55) for an [Hb] = 13 g/dl, obtaining *C* = 0.14 and β_SE-GE_ = 0.85. Importantly, *C* and β_SE-GE_, among other factors, are influenced by the extent of intravascular suppression.

**Figure 4.**
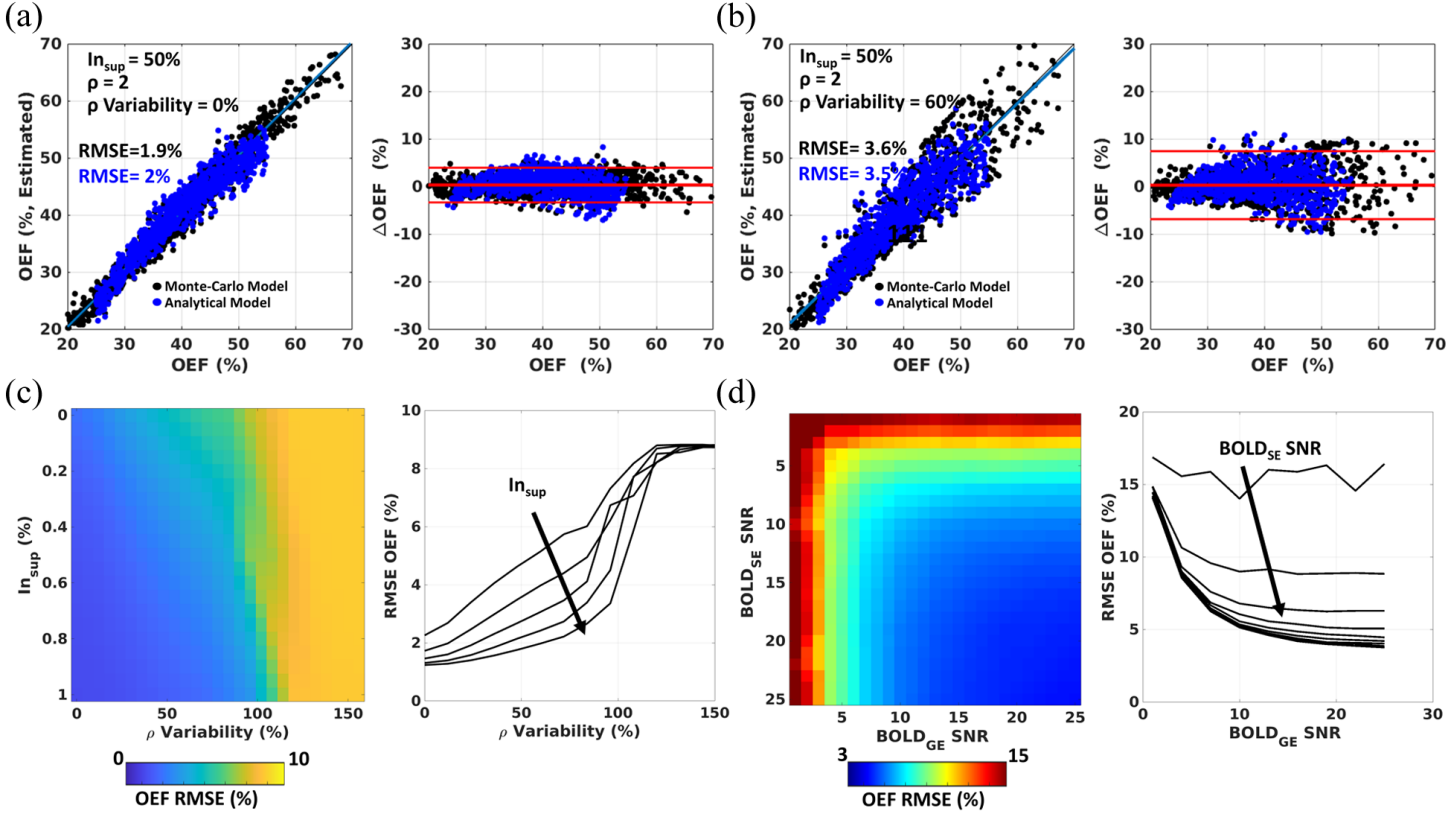
Correlation (left image) and Bland–Altman plot (right image) of the estimated OEF versus the simulated OEF using Monte Carlo modelling (black dots) or Davis analytical modelling (blue dots) for inversion and Monte Carlo forward modelling with In_sup_ = 50% (In_sup_ = level of intravascular signal suppression, between 0 and 1) and (a) fixing ρ = 2 (ρ = CBV_dHb_/CBV_cap_) or (b) considering ρ as a random variable with a variability of 60% (±3 σ/μ). OEF RMSE of Davis analytical modelling as a function of (c) In_sup_ and ρ variability, and as a function of (d) BOLD_GE_ SNR and BOLD_SE_ SNR, presented both as a bi-dimensional image (left image) and as a plot (right image). The arrows in the right images of subplots (c) and (d) depict increasing value of In_sup_ and BOLD_SE_ SNR, respectively.

Reports the results obtained with Monte Carlo forward modelling and inversion modelling implemented either by inverting the same Monte Carlo modelling or the Davis analytical modelling within the fitting OEF range used. Figure 4(a) shows correlation and Bland–Altman plots obtained with forward model simulations as presented in Figure 1 but with a fixed ρ = 2. Figure 4(b) reports the same simulation, but adding variability to ρ as reported in Figure 1. Intravascular signal suppression was fixed at 50% for both BOLD signals. The inversion models were able to estimate the OEF based on the BOLD_SE_/BOLD_GE_ signal changes ratio with an OEF RMSE of around 2% and 3.5%, respectively. Notably, the low RMSE obtained from inverting the Davis model fitted to the Monte Carlo simulations corroborates the validity of the analytical framework presented in the “Methods” section (equation (9)).

Figure 4(c) reports the OEF RMSE of the inversion using the Davis model as a function of In_sup_ and ρ variability. OEF RMSE increases as In_sup_ decreases and ρ variability increases, with the greater effect of ρ variability. With ρ variability up to 100%, the method delivers a RMSE <5%. Figure 4(d) reports the OEF RMSE as a function of BOLD_GE_ and BOLD_SE_ SNR for In_sup_ = 50% and ρ variability = 60%. The RMSE due to signal noise is below 5% when the SNR is above 8 for both BOLD_GE_ and BOLD_SE_ modulations.

### In vivo data

The average PetO_2_ and PetCO_2_ at rest were 112.7 ± 4.8 and 34.0 ± 3.0 mmHg, respectively. The BH task was successfully performed by all participants and induced a consistent modulation in PetCO_2_, reflecting in a modulation of CBF and BOLD signals.

**Figure 5.**
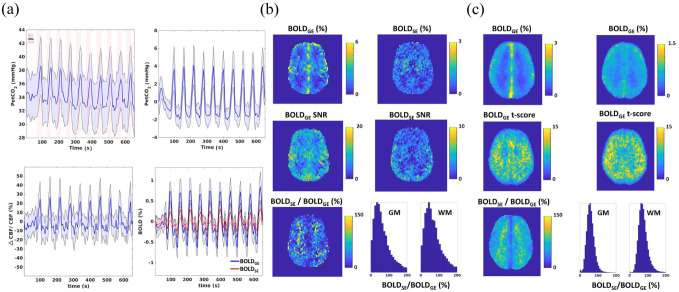
(a) Average raw PetCO_2_ trace across participants and filtered PetCO_2_ (top row) as well as average and filtered CBF and BOLD_GE_ and BOLD_SE_ modulations in the GM (bottom row), (b) representative maps for a participant of the study and (c) average maps in MNI space of BOLD_GE_ and BOLD_SE_ modulations (top row), of BOLD_GE_ and BOLD_SE_ SNR or *t*-score (middle row) and of BOLD_SE_/BOLD_GE_ signal changes ratio (bottom row, left image). The BOLD_SE_/BOLD_GE_ signal changes ratio distributions in GM and WM are also reported (bottom row, right image).

Figure 5(a) shows the average unfiltered (left) and filtered (right) PetCO_2_ traces (top row). The PetCO_2_ modulation (difference between the signal 95th and the fifth percentiles) was 6.9 ± 1.9 mmHg. Figure 5(a) also shows the average modulations in the GM for CBF and for BOLD_SE_ and BOLD_GE_, respectively (bottom row). The average GM modulation in CBF was 26% ± 16%, whereas the modulations in the BOLD signals were BOLD_GE_ = 1.50% ± 0.34% and BOLD_SE_ = 0.79% ± 0.24%. Figure 5(b) shows regional maps of the BOLD_GE_ and BOLD_SE_ modulations (top row), BOLD_GE_ and BOLD_SE_ SNR (middle row) and BOLD_SE_/BOLD_GE_ signal change ratio (bottom row, left image), for an exemplar study participant. The distribution of BOLD_SE_/BOLD_GE_ signal changes ratio in GM and WM is also reported (bottom row, right image). Figure 5(c) reports the same maps/plots as in Figure 5(b) but as average values across participants (with across subjects’ *t*-score maps substituting SNR). The subjects’ average BOLD_SE_/BOLD_GE_ signal changes ratio was 58.8% ± 21.9% in the GM and 74.5% ± 25.5% in the WM.

**Figure 6.**
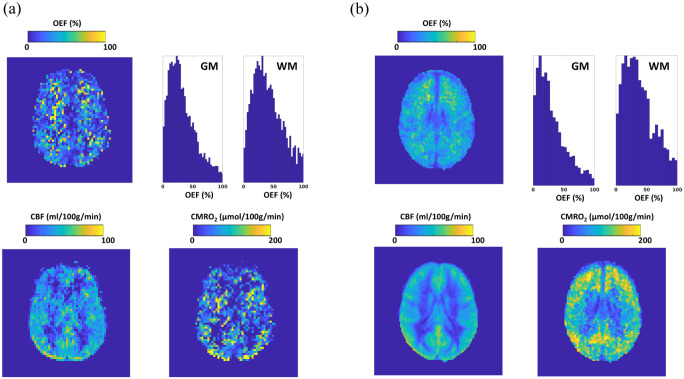
(a) Representative images for a participant of the study and (b) average maps in MNI space for OEF (distribution values in GM and WM are also reported), CBF and CMRO_2_.

Figure 6(a) shows a representative OEF map for a participant of the study computed from the BOLD_SE_/BOLD_GE_ signal changes ratio. CBF maps (computed with ASL at baseline) and CMRO_2_ maps are also displayed. Histograms of OEF values obtained in the GM and WM are also reported. Figure 6(b) reports the same maps/plots as in Figure 6(a) but as average values across participants in MNI space. Repeatability analysis of OEF maps delivered good voxelwise spatial correlations in GM and WM (across subjects’ *r* = 0.41 ± 0.09 for GM and *r* = 0.38 ± 0.09 for WM, respectively, both *p*’s < 10^−3^) and good-to-excellent correlations on a global basis (*r* = 0.84 for GM and *r* = 0.72 for WM, respectively, both *p*’s < 10^−3^, refer to Figure S1 in Supplementary Materials).

**Figure 7.**
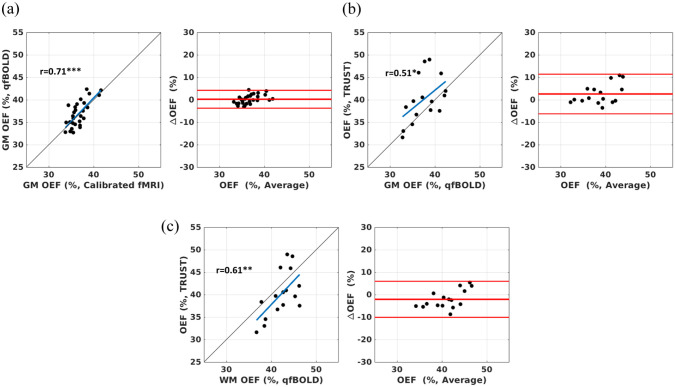
Scatterplots and Bland–Altmann plots comparing extracted global OEF values in the GM and WM with alternative approaches: (a) qfBOLD versus single calibration fMRI for GM and (b) TRUST versus qfBOLD in GM and (c) TRUST versus qfBOLD in WM.

Figure 7 reports scatterplots and Bland–Altman plots comparing global OEF values in the GM and WM obtained with qfBOLD with alternative approaches. The average OEF for qfBOLD was OEF = 37.0% ± 2.9% in the GM and OEF = 41.6% ± 2.9% in the WM with a significant correlation (*r* = 0.88, *p* < 10^−3^) and different average values between the two compartments (*p* < 10^−3^). Figure 6(a) compares the proposed method with the single calibration fMRI approach in the GM. The average GM OEF was OEF = 36.6% ± 2.0% for single calibration fMRI. A correlation of *r* = 0.71 (*p* < 10^−3^) was obtained in the GM with no bias between the approaches. Figure 7(b) and (c) compare the GM and WM OEF extracted with qfBOLD with the OEF evaluated in the SSS with TRUST in a subset of 16 subjects. The average OEF from TRUST was 40.1% ± 5.2%. The correlation coefficients in the GM and WM were *r* = 0.51 (*p* < 0.05) and *r* = 0.61 (*p* < 0.01), respectively. TRUST showed slightly larger OEF in the GM and smaller OEF in the WM compared to those obtained using the qfBOLD method (*p* < 0.05).

## Discussion

We have introduced a novel framework for mapping OEF at rest that relies on comparing (taking the ratio of) functional modulations in the gradient-echo and spin-echo BOLD signals (BOLD_GE_ and BOLD_SE_), both of which are concurrently acquired during isometabolic vasodilation. This method, termed quantitative functional BOLD (qfBOLD), differs from traditional qBOLD,^15 [Bibr bibr39-0271678X261453807]^ in both its approach and acquisition strategy. Traditional qBOLD quantifies OEF by comparing multi-TE GE and SE acquisitions, often relying on absolute estimations of *T*_2_*, *T*_2_ and *T*_2_′. In contrast, qfBOLD derives OEF from temporal fluctuations in *T*_2_* and *T*_2_, each estimated from BOLD_GE_ and BOLD_SE_ acquired at a single TE. As dHb is the only magnetic substance whose concentration oscillates over time, qfBOLD retains the advantage of selective sensitivity to baseline dHb as in cfMRI while offering several improvements.^21,24^ qfBOLD can decouple OEF from CBV_dHb_ with a single modulation of brain physiology and without additional assumptions, while eliminating the need for concurrent functional ASL recordings. This absence of ASL potentially greatly enhances SNR and may enable significant improvements in both temporal and spatial resolution, while potentially allowing extension of the method to WM.

Monte Carlo simulations of the BOLD_GE_ and BOLD_SE_ signal changes generated within a voxel with randomly oriented microvasculature during increases in CBF demonstrate the feasibility of the qfBOLD approach with the possibility to use the simplistic Davis model within the framework (Figures 1–4). Notably, the simulated BOLD_SE_/BOLD_GE_ signal changes ratio reveals a monotonic dependence on OEF (Figure 2(c)), which actually arises from a monotonic dependence on dHb levels in blood ([dHb], with a fixed concentration of haemoglobin in blood, [Hb] and arterial oxygen content, CaO_2_). Crucially, this relationship is nearly independent of baseline CBV_dHb_ and modulations in CBF (Figures 2(c) and 3), supporting the key features of the method, that is, its ability to decouple [dHb] and CBV_dHb_ relying only on vasodilation and without requiring functional CBF measures. The slope of this monotonic relationship is particularly pronounced under conditions of greater intravascular signal suppression.

Modelling and in vivo studies suggest that the intravascular signal accounts for around 30% of the BOLD_GE_ signal and 50% of the BOLD_SE_ signal,^27-31^ modifying the total signal dependence on [dHb] compared to pure extravascular effects,^26^ which primarily generate the method’s sensitivity to OEF. Clearly, the intravascular contribution can be modelled and accounted for, or alternatively, it can be suppressed to enhance the method’s sensitivity to OEF, with the added advantage of partially eliminating the effects of large vessels that the method ignores.

Indeed, the monotonic dependence of the signal ratio is also influenced by microvascular topology, as illustrated in Figure 3, where variability in the parameter ρ adds noise to the monotonic relationship between OEF and the BOLD_SE_/BOLD_GE_ ratio (Figure 3(b)). Nonetheless, the method’s ability to infer OEF is demonstrated in Figure 4, where inversion models achieved an RMSE in OEF estimation below 5% (Figure 4(a) and (b)). The inversion of the Davis model delivered sufficiently small RMSE up to ρ variability of around 100% (RMSE always below 5%) for almost any level of intravascular suppression (Figure 4(c)). This performance was contingent on maintaining a SNR >8 for both BOLD_GE_ and BOLD_SE_ signal changes following CBF modulation (Figure 4(d)), which is achievable in vivo (see Figure 5(b)). With respect to in vivo applications, the fitted Davis model was preferred for modelling inversion compared to the full Monte Carlo model, as simpler models are preferable in the presence of experimental noise.

The qfBOLD method exploiting the Davis model inversion was compared in vivo to a single cfMRI approach that we recently developed based on BOLD_GE_/ASL recordings acquired during BH.^7,25^ We acquired fMRI data during a BH task using a sequence that integrates functional BOLD_GE_, BOLD_SE_ and ASL. Additionally, the new method was also compared to global OEF measures in the sagittal sinus using a relaxometry-based method (TRUST).^12,63^

By comparing the average BOLD_SE_/BOLD_GE_ signal changes ratio in GM during the hypercapnic task (Figure 5(c)) with simulations (Figures 2–4), we determined that the developed sequence achieved ~50% intravascular suppression of the BOLD signals, consistent with an average OEF of ~40%. This estimated suppression is partly due to *T*1 weighting resulting from the short time interval between ASL and BOLD readouts, combined with the effects of gradients introduced around the spin-echo refocussing pulse. Moreover, the intravascular suppression model parameter may account for inaccuracies in the intravascular signal modelling, which relies on phenomenological equations.^49^

While the OEF maps generated by inverting the developed model appear plausible (Figure 6), it is important to note that macrovascular contamination is present in the maps, with clear regions where the BOLD_SE_ signal tends to be smaller than the BOLD_GE_ signal, leading to an underestimation of OEF. To mitigate the influence of errors induced by large vessels we focussed exclusively on voxels with a BOLD_SE_/BOLD_GE_ signal changes ratio above 30% when computing global values for correlation analysis with alternative methods.

The qfBOLD method yielded an average OEF = 37.0% ± 2.9% in GM, compatible with the single calibration fMRI approach (OEF = 36.6% ± 2.0%) and the TRUST method (OEF = 40.1% ± 5.2%). OEF values showed good within-session repeatability at a voxelwise level (spatial correlations: *r* = 0.41 ± 0.09 in GM and *r* = 0.38 ± 0.09 in WM, both *p*’s < 10^−3^), and good-to-excellent repeatability at a global level (*r* = 0.84 in GM and *r* = 0.72 in WM, *p*’s < 10^−3^). Importantly, OEF values measured with qfBOLD showed significant correlations with those estimated with the former alternative approaches on a global basis for GM (qfBOLD vs cfMRI *r* = 0.71, *p* < 10^−3^, qfBOLD vs TRUST *r* = 0.51, *p* < 0.05) and WM (qfBOLD vs TRUST *r* = 0.61, *p* < 0.01). WM exhibited higher average OEF (OEF = 41.6% ± 2.9%) compared to GM, probably induced by modelling limitations (see below). The global correlations we obtained between the OEF estimated with qfBOLD and that obtained through alternative approaches such as cfMRI and TRUST, strongly suggest the validity of the newly proposed approach.

However, several important assumptions in the vascular modelling warrant consideration. The assumption of isotropic vessel orientation, while predominantly valid in GM, may not hold in WM, where complex topology and anisotropic vasculature could contribute to an overestimation of OEF.^64–66^ Additionally, restricted diffusion in the extravascular space of WM may affect measurement accuracy, as water diffusion was assumed fixed in the model and tuned to GM. Finally, although the method focusses on modulations in *T*_2_ and *T*_2_* due to dHb, making it less susceptible than qBOLD to additional sources of magnetic susceptibility, it may retain residual sensitivity to WM tissue anisotropy and susceptibility-induced signal decay modifications, for example, those related to myelin.

While our results correlate well with two alternative methods further development is necessary. Future advancements in the method would require additional modelling and data acquisition improvements. Modelling may be refined by integrating realistic topologies of vascular compartments and variability in water diffusion, particularly for exploring WM. Experimentally, by eliminating ASL, the focus should be on acquiring high-quality BOLD_GE_ and BOLD_SE_ data with high spatiotemporal resolution and on maximising SNR for optimal method stability and repeatability. Moreover, macrovascular signal suppression and intravascular suppression of microvascular signals are warranted to reduce bias and variance.

The method repeatability between sessions and possibly MRI scanners should be assessed and, importantly, the approach should be tested in diseases where larger variability in vascular topology may decrease sensitivity to OEF. In addition, similarly to cfMRI approaches, the method requires a vascular reserve, which may be absent in diseases with compromised vasculature. In practice, when hypercapnia is induced via breath-holding, the task requires subject compliance.^25^ Another practical limitation of the approach, particularly for clinical settings, pertains to the need for acquiring end-tidal partial pressures. We used PetCO_2_ measures to derive the hypercapnic modulatory signal, and the PetO_2_ signal to derive baseline parameters of arterial oxygenation. However, an alternative approach that avoids gas recordings was previously presented by our group for cfMRI and it can be applied to qfBOLD. The approach is based on deriving the vasodilatory signal from the global BOLD signal.^25^ Moreover, baseline oxygenation may be fixed to standard values, as the error introduced in the OEF estimates is within a few percentage points.^42^ Nonetheless, hypercapnia itself may also introduce a bias in the measurements, as the assumption of isometabolism may not completely hold; some studies report that hypercapnia mildly reduces neural activity and CMRO_2_.^42,65–67^ However, this effect should introduce a smaller bias in the qfBOLD method compared to cfMRI, as changes in CMRO_2_ would similarly affect both BOLD_SE_ and BOLD_GE_.

The new qfBOLD method appears viable and holds promise for accurate mapping of brain oxygen extraction and consumption with MRI, offering advantages over current alternative methods such as qBOLD, QSM and cfMRI.

## Conclusion

We introduced a novel framework for OEF mapping with MRI, termed quantitative functional BOLD (qfBOLD). qfBOLD leverages the extravascular temporal dynamics of gradient-echo BOLD and spin-echo BOLD signals during isometabolic vasodilation. This innovative approach enhances sensitivity to baseline dHb compared to qBOLD. Furthermore, it effectively decouples OEF from CBV_dHb_ with a single modulation in brain physiology, without the need for concurrent ASL recordings, which are necessary in calibrated fMRI approaches. The avoidance of ASL improves SNR and allows for increased spatiotemporal resolution, enabling its application also in WM. While promising, the method’s reliance on specific vascular modelling assumptions, particularly in WM, highlights the need for further refinement in future applications. Ultimately, the qfBOLD method shows substantial potential for providing accurate and reliable assessments of brain oxidative metabolism. Further studies will be essential to optimise the method, for example, by maximising intravascular signal suppression, and explore its applicability across various pathological conditions.

## Supplemental Material

sj-docx-1-jcb-10.1177_0271678X261453807 – Supplemental material for Quantitative functional BOLD (qfBOLD): A combined gradient-echo and spin-echo framework for oxygen extraction fraction (OEF) mapping with functional MRI Supplemental material, sj-docx-1-jcb-10.1177_0271678X261453807 for Quantitative functional BOLD (qfBOLD): A combined gradient-echo and spin-echo framework for oxygen extraction fraction (OEF) mapping with functional MRI by Antonio Maria Chiarelli, Lucie Chalet, Sara Pomante, Davide Di Censo, Alessandra Caporale, Emma Biondetti, Fabrizio Fasano, Domenico Zaca, Giulia Rocco, Manuela Carriero, Francesca Graziano, Elizabeth Jane Fear, Maria Eugenia Caligiuri, Richard Geoffrey Wise and Michael Germuska in Journal of Cerebral Blood Flow & Metabolism

## References

[1] Senior AE. ATP synthesis by oxidative phosphorylation. Physiol Rev 1988; 68: 177–231. 2892214 10.1152/physrev.1988.68.1.177

[2] Magistretti P Allaman I. Brain energy metabolism. In: Pfaff DW (ed.) Neuroscience in the 21st century. New York, NY: Springer, 2013, pp.1591–1620.

[3] Lassen NA Christensen S. Physiology of cerebral blood flow. Br J Anaesth 1976; 48: 719–734. 7284 10.1093/bja/48.8.719

[4] Gjedde A. The pathway for oxygen in brain. APMIS Suppl 2003; 2003: 146–153. 12874967

[5] Gjedde A Poulsen PH Østergaard L . On the oxygenation of hemoglobin in the human brain. In: Eke A Delpy DT (eds.) Oxygen transport to tissue XXI. Boston, MA: Springer, 1999, pp.67–81. 10.1007/978-1-4615-4717-4_9 10659133

[6] Habler OP Messmer KFW . The physiology of oxygen transport. Transfus Sci 1997; 18: 425–435. 10175156 10.1016/S0955-3886(97)00041-6

[7] Chiarelli AM Germuska M Chandler H , et al. A flow-diffusion model of oxygen transport for quantitative mapping of cerebral metabolic rate of oxygen (CMRO_2_) with single gas calibrated fMRI. J Cereb Blood Flow Metab 2022; 42: 1192–1209. 35107026 10.1177/0271678X221077332 PMC9207485

[8] Rodgers ZB Detre JA Wehrli FW. MRI-based methods for quantification of the cerebral metabolic rate of oxygen. J Cereb Blood Flow Metab 2016; 36: 1165–1185. 27089912 10.1177/0271678X16643090 PMC4929705

[9] Fan AP An H Moradi F , et al. Quantification of brain oxygen extraction and metabolism with [^15^O]-gas PET: a technical review in the era of PET/MRI. Neuroimage 2020; 220: 117136. 32634594 10.1016/j.neuroimage.2020.117136 PMC7592419

[10] Alsop DC Detre JA Golay X , et al. Recommended implementation of arterial spin-labeled perfusion MRI for clinical applications: a consensus of the ISMRM perfusion study group and the European consortium for ASL in dementia. Magn Reson Med 2015; 73: 102–116. 24715426 10.1002/mrm.25197 PMC4190138

[11] Biondetti E Cho J Lee H. Cerebral oxygen metabolism from MRI susceptibility. Neuroimage 2023; 276: 120189. 37230206 10.1016/j.neuroimage.2023.120189 PMC10335841

[12] Lu H Ge Y. Quantitative evaluation of oxygenation in venous vessels using T_2_-relaxation-under-spin-tagging MRI. Magn Reson Med 2008; 60: 357–363. 18666116 10.1002/mrm.21627 PMC2587050

[13] Barhoum S Langham MC Magland JF , et al. Method for rapid MRI quantification of global cerebral metabolic rate of oxygen. J Cereb Blood Flow Metab 2015; 35: 1616–1622. 25966941 10.1038/jcbfm.2015.96 PMC4640312

[14] Liu EY Haist F Dubowitz DJ , et al. Cerebral blood volume changes during the BOLD post-stimulus undershoot measured with a combined normoxia/hyperoxia method. Neuroimage 2019; 185: 154–163. 30315908 10.1016/j.neuroimage.2018.10.032 PMC6292691

[15] He X Zhu M Yablonskiy DA. Validation of oxygen extraction fraction measurement by qBOLD technique. Magn Reson Med 2008; 60: 882–888. 18816808 10.1002/mrm.21719 PMC2812065

[16] Zhang J Liu T Gupta A , et al. Quantitative mapping of cerebral metabolic rate of oxygen (CMRO_2_) using quantitative susceptibility mapping (QSM). Magn Reson Med 2015; 74: 945–952. 25263499 10.1002/mrm.25463 PMC4375095

[17] Hametner S Endmayr V Deistung A , et al. The influence of brain iron and myelin on magnetic susceptibility and effective transverse relaxation – a biochemical and histological validation study. Neuroimage 2018; 179: 117–133. 29890327 10.1016/j.neuroimage.2018.06.007

[18] Duyn JH Schenck J. Contributions to magnetic susceptibility of brain tissue. NMR Biomed 2017; 30: e3546. 10.1002/nbm.3546 PMC5131875 27240118

[19] Davis TL Kwong KK Weisskoff RM , et al. Calibrated functional MRI: mapping the dynamics of oxidative metabolism. Proc Natl Acad Sci U S A 1998; 95: 1834–1839. 9465103 10.1073/pnas.95.4.1834 PMC19199

[20] Hoge RD. Calibrated fMRI. Neuroimage 2012; 62: 930–937. 22369993 10.1016/j.neuroimage.2012.02.022

[21] Germuska M Wise RG. Calibrated fMRI for mapping absolute CMRO_2_: practicalities and prospects. Neuroimage 2019; 187: 145–153. 29605580 10.1016/j.neuroimage.2018.03.068

[22] Bulte DP Kelly M Germuska M , et al. Quantitative measurement of cerebral physiology using respiratory-calibrated MRI. Neuroimage 2012; 60: 582–591. 22209811 10.1016/j.neuroimage.2011.12.017 PMC7100043

[23] Germuska M Bulte DP. MRI measurement of oxygen extraction fraction, mean vessel size and cerebral blood volume using serial hyperoxia and hypercapnia. Neuroimage 2014; 92: 132–142. 24531048 10.1016/j.neuroimage.2014.02.002

[24] Germuska M Chandler HL Stickland RC , et al. Dual-calibrated fMRI measurement of absolute cerebral metabolic rate of oxygen consumption and effective oxygen diffusivity. Neuroimage 2019; 184: 717–728. 30278214 10.1016/j.neuroimage.2018.09.035 PMC6264385

[25] Driver ID Chiarelli AM Chandler HL , et al. Breath-hold calibrated fMRI mapping of absolute cerebral metabolic rate of oxygen metabolism (CMRO_2_): an assessment of the accuracy and repeatability in a healthy adult population. Imaging Neurosci 2024; 2: 1–14. 10.1162/imag_a_00298 PMC7616461 40800477

[26] Ogawa S Menon RS Tank DW , et al. Functional brain mapping by blood oxygenation level-dependent contrast magnetic resonance imaging. A comparison of signal characteristics with a biophysical model. Biophys J 1993; 64: 803–812. 8386018 10.1016/S0006-3495(93)81441-3 PMC1262394

[27] Boxerman JL Bandettini PA Kwong KK , et al. The intravascular contribution to fMRI signal change: Monte Carlo modeling and diffusion-weighted studies in vivo. Magn Reson Med 1995; 34: 4–10. 7674897 10.1002/mrm.1910340103

[28] Boxerman JL Hamberg LM Rosen BR , et al. MR contrast due to intravascular magnetic susceptibility perturbations. Magn Reson Med 1995; 34: 555–566. 8524024 10.1002/mrm.1910340412

[29] Fujita N. Extravascular contribution of blood oxygenation level-dependent signal changes: a numerical analysis based on a vascular network model. Magn Reson Med 2001; 46: 723–734. 11590649 10.1002/mrm.1251

[30] Jochimsen TH Norris DG Mildner T , et al. Quantifying the intra- and extravascular contributions to spin-echo fMRI at 3 T. Magn Reson Med 2004; 52: 724–732. 15389950 10.1002/mrm.20221

[31] Zhong J Kennan RP Fulbright RK , et al. Quantification of intravascular and extravascular contributions to BOLD effects induced by alteration in oxygenation or intravascular contrast agents. Magn Reson Med 1998; 40: 526–536. 9771569 10.1002/mrm.1910400405

[32] Buxton RB. Introduction to functional magnetic resonance imaging: principles and techniques. Cambridge: Cambridge University Press, 2009.

[33] Li W Liu D van Zijl PCM , et al. Three-dimensional whole-brain mapping of cerebral blood volume and venous cerebral blood volume using Fourier transform-based velocity-selective pulse trains. Magn Reson Med 2021; 86: 1420–1433. 33955583 10.1002/mrm.28815 PMC8527552

[34] Gould IG Tsai P Kleinfeld D , et al. The capillary bed offers the largest hemodynamic resistance to the cortical blood supply. J Cereb Blood Flow Metab 2017; 37: 52–68. 27780904 10.1177/0271678X16671146 PMC5363755

[35] Brittain JF McCabe C Khatun H , et al. An MRI-histological study of white matter in stroke-free SHRSP. J Cereb Blood Flow Metab 2013; 33: 760–763. 23403376 10.1038/jcbfm.2013.14 PMC3652693

[36] Troprès I Grimault S Vaeth A , et al. Vessel size imaging. Magn Reson Med 2001; 45: 397–408. 11241696 10.1002/1522-2594(200103)45:3<397::aid-mrm1052>3.0.co;2-3

[37] Shen Y Ahearn T Clemence M , et al. Magnetic resonance imaging of the mean venous vessel size in the human brain using transient hyperoxia. Neuroimage 2011; 55: 1063–1067. 21224003 10.1016/j.neuroimage.2010.12.084

[38] Lemasson B Valable S Farion R , et al. In vivo imaging of vessel diameter, size, and density: a comparative study between MRI and histology. Magn Reson Med 2013; 69: 18–26. 22431289 10.1002/mrm.24218

[bibr39-0271678X261453807] Germuska MA Meakin JA Bulte DP. The influence of noise on bold-mediated vessel size imaging analysis methods. J Cereb Blood Flow Metab 2013; 33: 1857–1863. 23942365 10.1038/jcbfm.2013.141 PMC3851896

[40] Grubb RL Raichle ME Eichling JO , et al. The effects of changes in PaCO_2_ cerebral blood volume, blood flow, and vascular mean transit time. Stroke 1974; 5: 630–639. 4472361 10.1161/01.str.5.5.630

[41] Leung TS Tachtsidis I Tisdall MM , et al. Estimating a modified Grubb’s exponent in healthy human brains with near infrared spectroscopy and transcranial Doppler. Physiol Meas 2009; 30: 1–12. 19039165 10.1088/0967-3334/30/1/001

[42] Chiarelli AM Germuska M Di Censo D , et al. Multiparametric mapping of brain oxygen consumption with resting state calibrated functional MRI. Neuroimage 2025; 320: 121465. 40975145 10.1016/j.neuroimage.2025.121465

[43] Chiacchiaretta P Romani GL Ferretti A. Sensitivity of BOLD response to increasing visual contrast: spin echo versus gradient echo EPI. Neuroimage 2013; 82: 35–43. 23707589 10.1016/j.neuroimage.2013.05.069

[44] Uludağ K Müller-Bierl B Uğurbil K. An integrative model for neuronal activity-induced signal changes for gradient and spin echo functional imaging. Neuroimage 2009; 48: 150–165. 19481163 10.1016/j.neuroimage.2009.05.051

[45] Jochimsen TH von Mengershausen M. ODIN – object-oriented development interface for NMR. J Magn Reson 2004; 170: 67–78. 15324759 10.1016/j.jmr.2004.05.021

[46] Insiripong S Supattarobol T Jetsrisuparb A. Comparison of hematocrit/hemoglobin ratios in subjects with alpha-thalassemia, with subjects having chronic kidney disease and normal subjects. Southeast Asian J Trop Med Public Health 2013; 44: 707–711. 24050107

[47] Jensen JH Chandra R. Strong field behavior of the NMR signal from magnetically heterogeneous tissues. Magn Reson Med 2000; 43: 226–236. 10680686 10.1002/(sici)1522-2594(200002)43:2<226::aid-mrm9>3.0.co;2-p

[48] Bihan DL Warach SJ. Diffusion and perfusion magnetic resonance imaging: applications to functional MRI. J Comput Assist Tomogr 1995; 19: 844.

[49] Zhao JM Clingman CS Närväinen MJ , et al. Oxygenation and hematocrit dependence of transverse relaxation rates of blood at 3T. Magn Reson Med 2007; 58: 592–597. 17763354 10.1002/mrm.21342

[50] Schmithorst VJ Hernandez-Garcia L Vannest J , et al. Optimized simultaneous ASL and BOLD functional imaging of the whole brain. J Magn Reson Imaging 2014; 39: 1104–1117. 24115454 10.1002/jmri.24273 PMC3964152

[51] Okell TW Chappell MA Kelly ME , et al. Cerebral blood flow quantification using vessel-encoded arterial spin labeling. J Cereb Blood Flow Metab 2013; 33: 1716–1724. 23921895 10.1038/jcbfm.2013.129 PMC3824178

[52] Murphy K Harris AD Wise RG. Robustly measuring vascular reactivity differences with breath-hold: normalising stimulus-evoked and resting state BOLD fMRI data. Neuroimage 2011; 54: 369–379. 20682354 10.1016/j.neuroimage.2010.07.059

[53] Marques JP Kober T Krueger G , et al. MP2RAGE, a self bias-field corrected sequence for improved segmentation and T1-mapping at high field. Neuroimage 2010; 49: 1271–1281. 19819338 10.1016/j.neuroimage.2009.10.002

[54] Takano Y Sakamoto O Kiyofuji C , et al. A comparison of the end-tidal CO_2_ measured by portable capnometer and the arterial PCO_2_ in spontaneously breathing patients. Respir Med 2003; 97: 476–481. 12735663 10.1053/rmed.2002.1468

[55] Tustison NJ Cook PA Holbrook AJ , et al. The ANTsX ecosystem for quantitative biological and medical imaging. Sci Rep 2021; 11: 9068. 33907199 10.1038/s41598-021-87564-6 PMC8079440

[56] Jenkinson M Beckmann CF Behrens TEJ , et al. FSL. Neuroimage 2012; 62: 782–790. 21979382 10.1016/j.neuroimage.2011.09.015

[57] Zhang Y Brady M Smith S. Segmentation of brain MR images through a hidden Markov random field model and the expectation-maximization algorithm. IEEE Trans Med Imaging 2001; 20: 45–57. 11293691 10.1109/42.906424

[58] Andersson JLR Skare S Ashburner J. How to correct susceptibility distortions in spin-echo echo-planar images: application to diffusion tensor imaging. Neuroimage 2003; 20: 870–888. 14568458 10.1016/S1053-8119(03)00336-7

[59] Tustison NJ Avants BB Cook PA , et al. N4ITK: improved N3 bias correction. IEEE Trans Med Imaging 2010; 29: 1310–1320. 20378467 10.1109/TMI.2010.2046908 PMC3071855

[60] Wang Z Aguirre GK Rao H , et al. Empirical optimization of ASL data analysis using an ASL data processing toolbox: ASLtbx. Magn Reson Imaging 2008; 26: 261–269. 17826940 10.1016/j.mri.2007.07.003 PMC2268990

[61] Mutsaerts HJMM Steketee RME Heijtel DFR , et al. Inter-vendor reproducibility of pseudo-continuous arterial spin labeling at 3 Tesla. PLoS One 2014; 9: e104108. 10.1371/journal.pone.0104108 PMC4121318 25090654

[62] Penny WD Friston KJ Ashburner JT , et al. Statistical parametric mapping: the analysis of functional brain images. London, UK: Elsevier, 2011.

[63] Lu H Xu F Grgac K , et al. Calibration and validation of TRUST MRI for the estimation of cerebral blood oxygenation. Magn Reson Med 2012; 67: 42–49. 21590721 10.1002/mrm.22970 PMC3158970

[64] Nonaka H Akima M Hatori T , et al. Microvasculature of the human cerebral white matter: Arteries of the deep white matter. Neuropathology 2003; 23: 111–118. 12777099 10.1046/j.1440-1789.2003.00486.x

[65] Schilling KG Newton A Tax CMW , et al. The relationship of white matter tract orientation to vascular geometry in the human brain. Sci Rep 2025; 15: 18396. 40419741 10.1038/s41598-025-99724-z PMC12106635

[66] Doucette J Wei L Hernández-Torres E , et al. Rapid solution of the Bloch-Torrey equation in anisotropic tissue: application to dynamic susceptibility contrast MRI of cerebral white matter. NeuroImage 2019; 185: 198–207. 30332614 10.1016/j.neuroimage.2018.10.035

[67] Baas KPA Vu C Shen J , et al. Venous blood oxygenation measurements using TRUST and T_2_-TRIR MRI during hypoxic and hypercapnic gas challenges. J Magn Reson Imaging 2023; 58: 1903–1914. 37092724 10.1002/jmri.28744

[68] Deckers PT Bhogal AA Dijsselhof MB , et al. Hemodynamic and metabolic changes during hypercapnia with normoxia and hyperoxia using pCASL and TRUST MRI in healthy adults. J Cereb Blood Flow Metab 2022; 42: 861–875. 34851757 10.1177/0271678X211064572 PMC9014679

[69] James S Sanggaard S Akif A , et al. Spatiotemporal features of neurovascular (un)coupling with stimulus-induced activity and hypercapnia challenge in cerebral cortex and olfactory bulb. J Cereb Blood Flow Metab 2023; 43: 1891–1904. 37340791 10.1177/0271678X231183887 PMC10676132

