# Supplementary material for: Quantitative functional BOLD (qfBOLD): A combined gradient-echo and spin-echo framework for oxygen extraction fraction (OEF) mapping with functional MRI
Source: J Cereb Blood Flow Metab. 2026 May 28:0271678X261453807. Online ahead of print. doi: 10.1177/0271678X261453807 (PMC13379522; doi:10.1177/0271678X261453807)
Supplement: sj-docx-1-jcb-10.1177_0271678X261453807 – Supplemental material for Quantitative functional BOLD (qfBOLD): A combined gradient-echo and spin-echo framework for oxygen extraction fraction (OEF) mapping with functional MRI [file sj-docx-1-jcb-10.1177_0271678X261453807.docx]

**Supplementary Materials.**

**Motion correction pipeline for the functional data.**

An ad-hoc motion correction pipeline was implemented to minimize the effect of ASL tag and control alternation. The three BH fMRI time courses (ASL, GE-BOLD and SE-BOLD) were split based on tag and control and rigidly registered to the respective first volumes using FLIRT from FSL.^62–64^ Then, the first volume of the control images was registered to the first volume of the tag images using transformations computed only on GE-BOLD weighting (which showed minimal perfusion weighting and had higher SNR compared to SE-BOLD). All control volumes for ASL and SE-BOLD for each weighting were registered to tags using the derived transformation.

**Table S1.** MRI protocols and acquisition parameters

| **Sequence** | **MP2RAGE** | **(pC)ASL DEXI GE+SE EPI** | **TRUST** |
| --- | --- | --- | --- |
| Resolution [mm^3^] | 1.0 x 1.0 x 1.0 | 3.4 x 3.4 x 7.0 | 3.4 x 3.4 x 5.0 |
| Matrix size [voxels] | 176 x 256 x 256 | 64 x 64 x 14 | 64 x 64 |
| Slice gap [mm] | - | 30% | - |
| Pixel Bandwidth [Hz/Px] | 179 | 2112 | 3256 |
| Echo Time (TE) [ms] | 3.58 | TE1 = 10  TE2 = 30  TE3 = 85 | eTE=0  eTE=40  eTE=80  eTE=160 |
| Repetition Time (TR) [ms] | 5000 | 5000 | 3000 |
| Inversion Time (TI) [ms] | TI1 = 700  TI2 = 2500 | - | 1020 |
| Labelling duration (τ) [ms] | - | 1500 | - |
| Post Labelling Delay (PLD) [ms] | - | 1500 | - |
| GRAPPA acceleration | 3 | 3 | 3 |

**Within-session repeatability analysis**

Figure S1 shows the within-session repeatability of OEF estimates obtained from the qfBOLD approach applied to the first and last five breath-holds (BH) of the hypercapnic task. Subplots a) and b) show, respectively, the distribution of subject-wise spatial correlations for grey matter (GM) and white matter (WM). A t-test applied to the Fisher-transformed correlations indicated that the average spatial correlation was significantly above 0 (p<10^-3^). Subplots c and d present correlation and Bland-Altman plots for GM and WM, respectively, considering the two OEF estimates. High and significant associations are evident in the correlation plots (r=0.84 for GM and r 0.72 for WM, both p’s < 10^-3^), whereas the Bland-Altman plots show no significant bias.

**
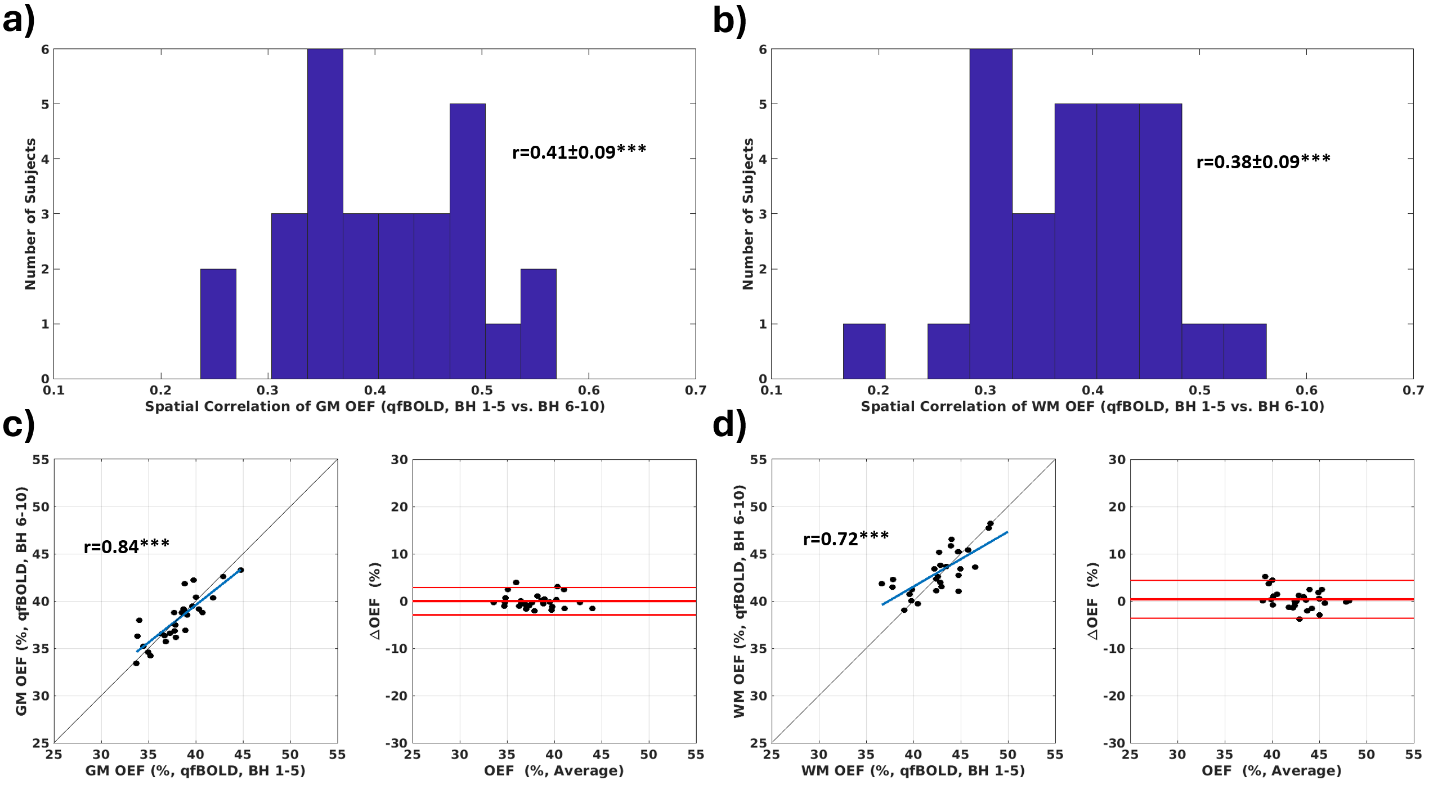
**

**Figure S1.** qfBOLD within-session repeatability analysis (BH 1-5 vs. BH 6-10). Distribution across subjects of voxelwise spatial correlation of OEFs for **a)** GM and **b)** WM. Comparison (correlation and Bland-Altman plots) of global OEF values for **c)** GM and **d)** WM. *** p<10^-3^
